# Does Chronic Pancreatitis in Growing Pigs Lead to Articular Cartilage Degradation and Alterations in Subchondral Bone?

**DOI:** 10.3390/ijms25041989

**Published:** 2024-02-06

**Authors:** Ewa Tomaszewska, Monika Hułas-Stasiak, Piotr Dobrowolski, Małgorzata Świątkiewicz, Siemowit Muszyński, Agnieszka Tomczyk-Warunek, Tomasz Blicharski, Janine Donaldson, Marcin B. Arciszewski, Michał Świetlicki, Iwona Puzio, Joanna Bonior

**Affiliations:** 1 Department of Animal Physiology, Faculty of Veterinary Medicine, University of Life Sciences in Lublin, 20-950 Lublin, Poland; iwona.puzio@up.lublin.pl; 2 Department of Functional Anatomy and Cytobiology, Faculty of Biology and Biotechnology, Maria Curie-Sklodowska University, 20-033 Lublin, Poland; monhul@o2.pl (M.H.-S.); piotr.dobrowolski@umcs.lublin.pl (P.D.); 3 Department of Animal Nutrition and Feed Science, National Research Institute of Animal Production, 32-083 Balice, Poland; malgorzata.swiatkiewicz@iz.edu.pl; 4 Department of Biophysics, Faculty of Environmental Biology, University of Life Sciences in Lublin, 20-950 Lublin, Poland; siemowit.muszynski@up.lublin.pl; 5 Laboratory of Locomotor System Research, Department of Rehabilitation and Physiotherapy, Medical University in Lublin, 20-090 Lublin, Poland; agnieszka.tomczyk-warunek@umlub.pl; 6 Department of Orthopaedics and Rehabilitation, Medical University in Lublin, 20-090 Lublin, Poland; blicharski@vp.pl; 7 School of Physiology, Faculty of Health Sciences, University of the Witwatersrand, Parktown, Johannesburg 2193, South Africa; janine.donaldson@wits.ac.za; 8 Department of Animal Anatomy and Histology, University of Life Sciences in Lublin, 20-950 Lublin, Poland; mb.arciszewski@wp.pl; 9 Department of Applied Physics, Faculty of Mechanical Engineering, Lublin University of Technology, 20-618 Lublin, Poland; m.swietlicki@pollub.pl; 10 Department of Medical Physiology, Chair of Biomedical Sciences, Institute of Physiotherapy, Faculty of Health Sciences, Jagiellonian University Medical College, 31-501 Cracow, Poland; joanna.bonior@uj.edu.pl

**Keywords:** pig, chronic pancreatitis, articular cartilage, cytokines

## Abstract

Chronic pancreatitis (CP), a progressive inflammatory disease, poses diagnostic challenges due to its initially asymptomatic nature. While CP’s impact on exocrine and endocrine functions is well-recognized, its potential influence on other body systems, particularly in young individuals, remains underexplored. This study investigates the hypothesis that CP in growing pigs leads to alterations in articular cartilage and subchondral bone, potentially contributing to osteoarthritis (OA) development. Utilizing a pig model of cerulein-induced CP, we examined the structural and compositional changes in subchondral bone, articular cartilage, and synovial fluid. Histological analyses, including Picrosirius Red and Safranin-O staining, were employed alongside immuno-histochemistry and Western blotting techniques. Our findings reveal significant changes in the subchondral bone, including reduced bone volume and alterations in collagen fiber composition. Articular cartilage in CP pigs exhibited decreased proteoglycan content and alterations in key proteins such as MMP-13 and TGF-β1, indicative of early cartilage degradation. These changes suggest a link between CP and musculoskeletal alterations, underscoring the need for further research into CP’s systemic effects. Our study provides foundational insights into the relationship between CP and skeletal health, potentially guiding future pediatric healthcare strategies for early CP diagnosis and management.

## 1. Introduction

Chronic pancreatitis (CP) poses a considerable diagnostic challenge due to its prolonged asymptomatic phase, making it difficult to detect early. This irreversible and progressive inflammatory disease impacts both the exocrine and endocrine functions of the pancreas. When over 90% of the pancreas is damaged, it results in pancreatic insufficiency [1]. CP is a well-known causative factor of the development of systemic inflammation, characterized by a rapid progression that weakens the entire organism. As a complex disease, CP extends its multifaceted influence on various systems and organs in both adults and children [2,3]. Pancreatitis is a prevalent concern in gastroenterology, affecting individuals across different age groups, from children to young adults [2,4,5,6].

Our previous research established a pig model of cerulein-induced chronic pancreatitis (CP), replicating repetitive episodes of short acute pancreatitis that resulted in organ damage. This model exhibited symptoms similar to those observed in humans during clinical observations. Moreover, an increase in pro-inflammatory cytokines in blood serum (interleukin 1 beta (IL-1β), interleukin 6 (IL-6), and tumor necrosis factor alpha (TNF-α)), as well as inflammatory indicators (C-reactive protein (CRP), lactate dehydrogenase (LDH), gamma-glutamyltransferase (GGTP), superoxide dismutase (SOD), and reduced glutathione (GSH)) were observed in the pigs, confirming systemic inflammation [7]. There is a clear association between chronic systemic inflammation and osteoporosis (OP), a systemic bone metabolic disorder characterized by deteriorating bone microarchitecture and increased bone vulnerability [8,9] which contributes to accelerated bone loss and an increased risk of fractures in affected individuals [10,11]. Additionally, osteoporosis may coexist with conditions such as osteoarthritis [12,13], a complex disease that leads to joint pain, reduced joint mobility, and impaired joint function [14,15]. Chronic systemic inflammation, often characterized by elevated levels of inflammatory markers like interleukin-6 (IL-6) and tumor necrosis factor-alpha (TNF-α), has been associated with the development and progression of osteoarthritis (OA), one of the prevailing musculoskeletal disorders with clinical manifestations involving pain and functional limitations, encompassing joint stiffness and dysfunction, and the gradual degenerative deterioration of cartilage, accompanied by synovitis as the primary morphological feature [16]. This chronic, low-grade inflammation can disrupt cartilage homeostasis, beginning with cartilage matrix damage [17,18]. Moreover, systemic inflammation can trigger heightened production of reactive oxygen species and induce oxidative stress, adversely impacting the health of both bone and cartilage. Oxidative stress is recognized as a contributing factor to the development of cartilage pathology [19]. Current understanding indicates that the disease extends beyond the impact on articular cartilage alone; it encompasses all tissues, both within and outside the joint. This results in the emergence of low-grade inflammation in the menisci, ligaments, periarticular muscles, joint capsule, synovial membrane, and subchondral bone [20] Subchondral bone lesions play a specific role in initiating the degeneration of articular cartilage and vessel erosion. This is attributed to direct molecular signaling that establishes a connection between cartilage and subchondral bone [21,22]. Furthermore, OA is not exclusive to the elderly population; it can also manifest in younger individuals [23,24]. The presence of OA is linked to adverse outcomes in children. Despite the growing research interest in CP and juvenile osteoarthritis, there has been no animal study conducted on this emerging concept in pediatric medicine.

Thus, we hypothesized that experimental CP in young animals, leading to systemic inflammation [7], can result in the development of OA over time. The goal of the study was to analyze the basal structure of subchondral bone and articular cartilage, pro-inflammatory profile, and the content of matrix metalloproteinase-13 (MMP-13) in synovial fluid, MMP-13 and transforming growth factor β1 (TGF-β1) expression in articular cartilage in growing piglets with CP.

## 2. Results

### 2.1. Subchondral Bone

**Figure 1:**
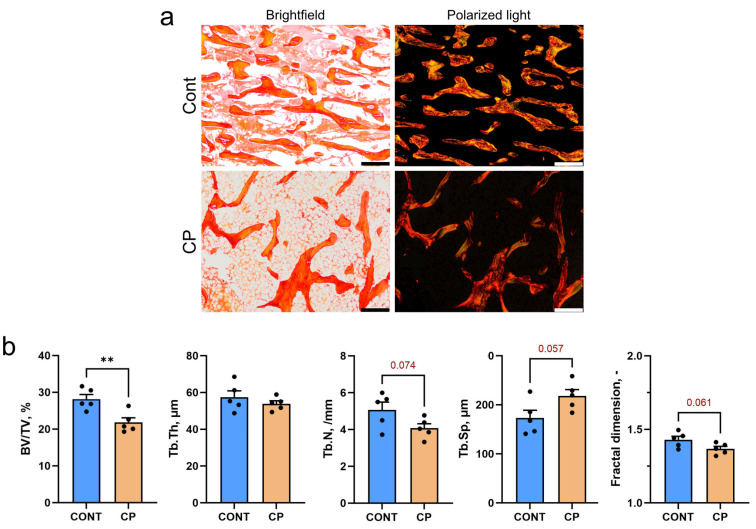
(**a**) Representative images of the PSR-stained sections of subchondral bone trabeculae in the distal femur of control (CONT) and chronic pancreatitis (CP) pigs. Observations were carried out using both brightfield and polarized light microscopy. All scale bars show 200 μm. (**b**) Histomorphometric parameters of bone trabeculae: volumetric bone fraction (BV/TV), trabecular thickness (Tb.Th), trabecular number (Tb.N), trabecular spacing (Tb.Sp), fractal dimension. Measurements were performed on two histological sections per animal, with four randomly selected areas of the subchondral bone evaluated per section, yielding a total of eight measurements per animal. Bars represent mean values ± standard error (n = 5 pigs per group). Statistical significance: ** *p* < 0.01. Trends towards statistical significance (*p* < 0.1) are also indicated.

Figure 1a displays representative images of Picrosirius red (PSR)-stained sections of subchondral bone trabeculae in the distal femur of control (CONT) and cerulein-pancreatitis (CP) groups. The images, observed under polarized light, highlight differences between large (red–orange) and thin (green) collagen fibers. In the cerulein-induced CP group there was a decrease in the amount of loosely packed thin collagen fibers (Figure 1a). The induction of CP led to a significant reduction in bone volume (BV/TV), while there was a trend (*p* < 0.1) for decreased trabecular number (Tb.N) and fractal dimension. Additionally, there was a trend for an increase in trabecular space (Tb.Sp) in the CP group compared to the control (Figure 1b).

### 2.2. Articular Cartilage

**Figure 2:**
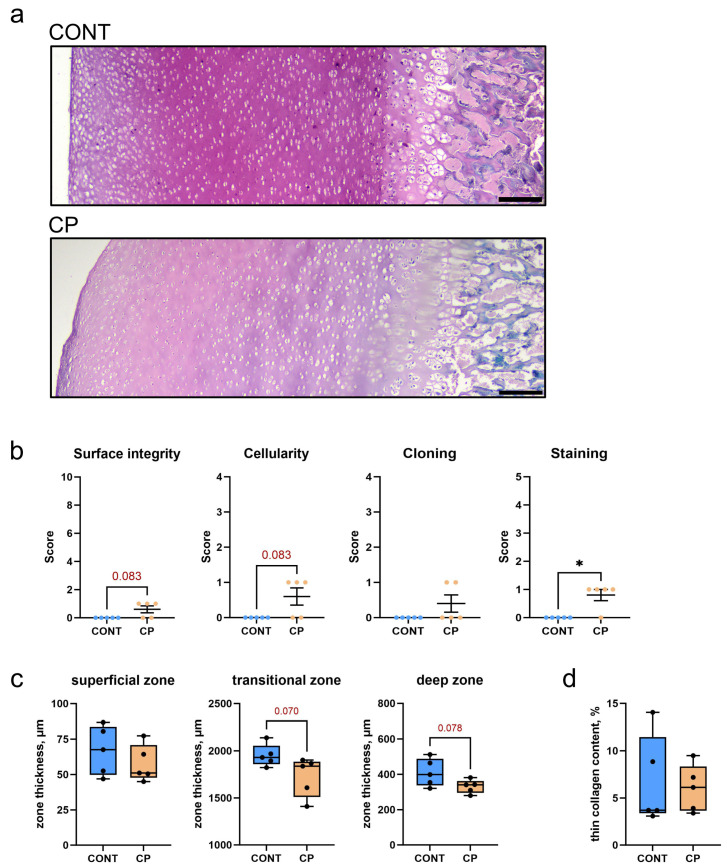
(**a**) Representative Safranin-O-stained sections of articular cartilage in the distal femur from control (CONT) and chronic pancreatitis (CP) pigs. Scale bars show 200 μm. (**b**) Summary of experimental group comparisons of cartilage Mankin grading scale protocols. Plots indicate mean values ± standard error. (**c**) Histomorphometric parameters of the zones of distal femoral articular cartilage. (**d**) Content of immature, thin collagen fibers in the articular cartilage. Measurements were performed on two histological sections per animal, with at least ten randomly selected areas cartilage. Box and whisker plots represent mean values (line within the box), interquartile range (box), and the max min range (whiskers) (n = 5 pigs per group). Statistical significance is denoted as * *p* < 0.05 (the Mann–Whitney U test). Trends towards statistical significance (*p* < 0.1) are also indicated.

Figure 2a illustrates Safranin-O-stained articular cartilage, highlighting notable proteoglycan content differences between the control group and piglets with cerulein-induced CP. In the control group, the articular cartilage exhibited a robust pink color, consistent across the transitional and deep zones, with slight brightening in the superficial zone. In contrast, the CP group showed diminished Safranin-O-positive proteoglycan staining, characterized by a uniformly lighter pink color that faded near the subchondral bone.

A detailed Mankin-type scoring system was employed to evaluate the extent of osteoarthritis in the knees of the CP group (Figure 2b). This included a statistically significant reduction in Safranin-O staining, and trends toward surface irregularities and a decrease in cellularity compared to the control. Collectively, these findings suggest mild cartilage degeneration associated with CP, with the total Mankin score for the CP group being a median of 2 and a mean of 2.4.

Figure 2c presents the histomorphometric analysis of the distal femoral articular cartilage in both the control and CP groups. While the superficial zone’s thickness was similar between groups, there was a trend for a reduced thickness of the transitional and deep zones, suggesting a potential thinning in these areas.

Figure 2d, shows the analysis of the PSR-stained section of articular cartilage, and reveals no significant differences in the content of thin (immature) and large (mature) collagen fibers between the control pigs and those with CP.

**Figure 3:**
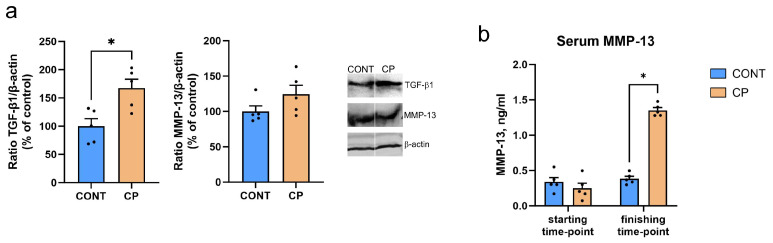
(**a**) Western blot analysis of TGF-β1 and MMP-13 protein levels in the articular cartilage of the distal femur in the control (CONT) and chronic pancreatitis (CP) pig groups. (**b**) Blood serum MMP-13 concentrations at the beginning and end of the experimental period. Uncropped original Western blot membranes of can be found in Supplementary Figure S1. Western blot analysis was performed with n = 2 technical replicates for each biological sample. For serum MMP-13 assay each sample underwent n = 3 technical replicates. Bars represent mean values ± standard error (n = 5 pigs per group). Statistical significance: * *p* < 0.05.

Western blot analysis of TGF-β1 and MMP-13 protein expression in articular cartilage, as shown in Figure 3a, revealed that TGF-β1 expression was significantly higher in the articular cartilage of pigs with CP, compared to the control group. In contrast, MMP-13 expression in articular cartilage was not significantly different between groups. However, the increase in blood serum MMP-13 concentrations was observed at the end of the experiment, as depicted in Figure 3b.

### 2.3. Immunohistochemistry of Articular Cartilage

**Figure 4:**
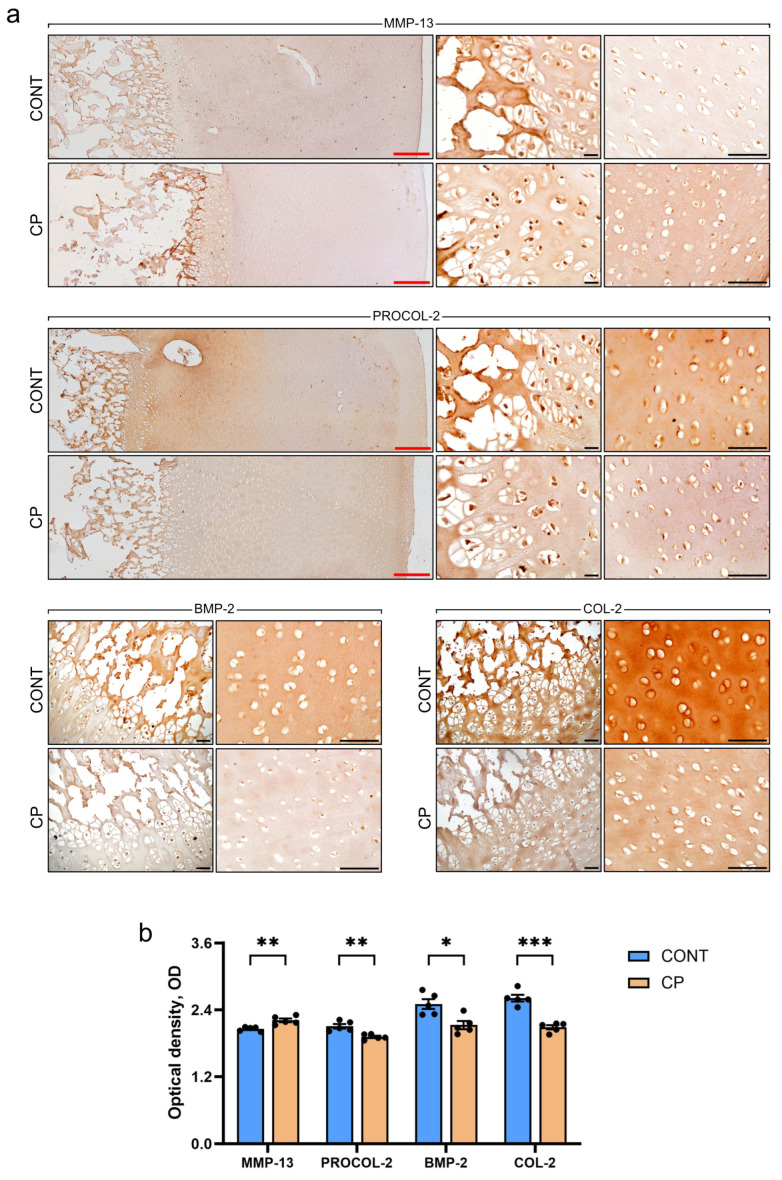
(**a**) Representative immunohistochemical (IHC) staining for MMP-13, PROCOL-2, BMP-2, and COL-2 in the articular cartilage of the distal femur from control (CONT) and chronic pancreatitis (CP) pigs. Red scale bars represent 200 μm, while black scale bars represent 50 μm. (**b**) Bar graphs illustrating the optical density (OD) of expression for the analyzed proteins. Measurements were conducted on two histological samples per animal in at least ten randomly selected areas. Bars indicate mean values ± standard error (n = 5 pigs per group). Statistical significance is denoted as * *p* < 0.05; ** *p* < 0.01; *** *p* < 0.001.

Figure 4a shows representative images, while Figure 4b presents the results of the quantitative analysis of optical density (OD) in immunohistochemical (IHC) staining. Generally, the immunoreactivity (IR) of chondrocytes was observed in all zones of articular cartilage. Despite no differences in MMP-13 expression detected in the Western blot analysis, the IHC analysis (which included the subchondral bone area) revealed a significant increase in the OD of MMP-13 IR chondrocytes in the CP group. There was a notable heterogeneity, with strong positive signals, in the subchondral bone, diminishing towards the superficial zone. In contrast, control pigs exhibited weak MMP-13 staining in the subchondral bone, consistent across all zones of articular cartilage. To address the imbalance in extracellular matrix (ECM) synthesis and degradation, a critical factor in cartilage damage, bone morphogenetic protein 2 (BMP-2) protein expression levels in the articular cartilage were evaluated. A decrease in the OD of BMP-2 IR was observed in CP pigs. Furthermore, the intensity of type II procollagen (PROCOL-2) and collagen (COL-2), essential constituents of the cartilage matrix, were evaluated. The OD of PROCOL-2 and COL-2 immunoreactivity was decreased in articular cartilage from pigs with CP.

**Figure 5:**
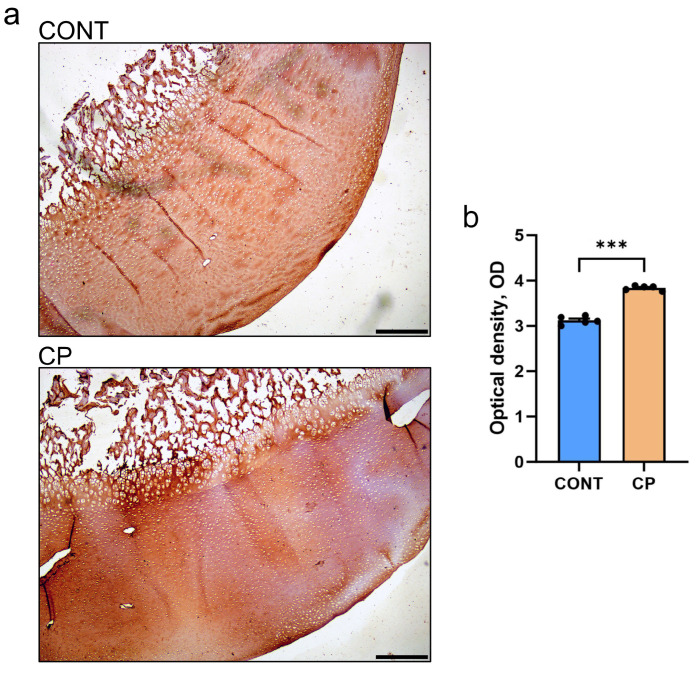
(**a**) Representative immunohistochemical (IHC) staining for COMP in the articular cartilage of the distal femur in control (CONT) and chronic pancreatitis (CP) pigs. Scale bars show 400 μm. (**b**) Bar graph illustrating the optical density (OD) of expression for the analyzed protein. Measurements were conducted on two histological samples per animal in at least ten randomly selected areas. Bars indicate mean values ± standard error (n = 5 pigs per group). Statistical significance is denoted as *** *p* < 0.001.

Cartilage oligomeric matrix protein (COMP), a key component of the cartilage matrix and biomarker of cartilage degeneration, was also evaluated (Figure 5). IHC staining for COMP in healthy cartilage showed weak staining in territorial and interterritorial matrix areas, while in CP pigs, COMP staining was more pronounced in the same areas (Figure 5a). A semiquantitative densitometric analysis showed that COMP immunoreactivity was increased in CP pigs (Figure 5b).

**Figure 6:**
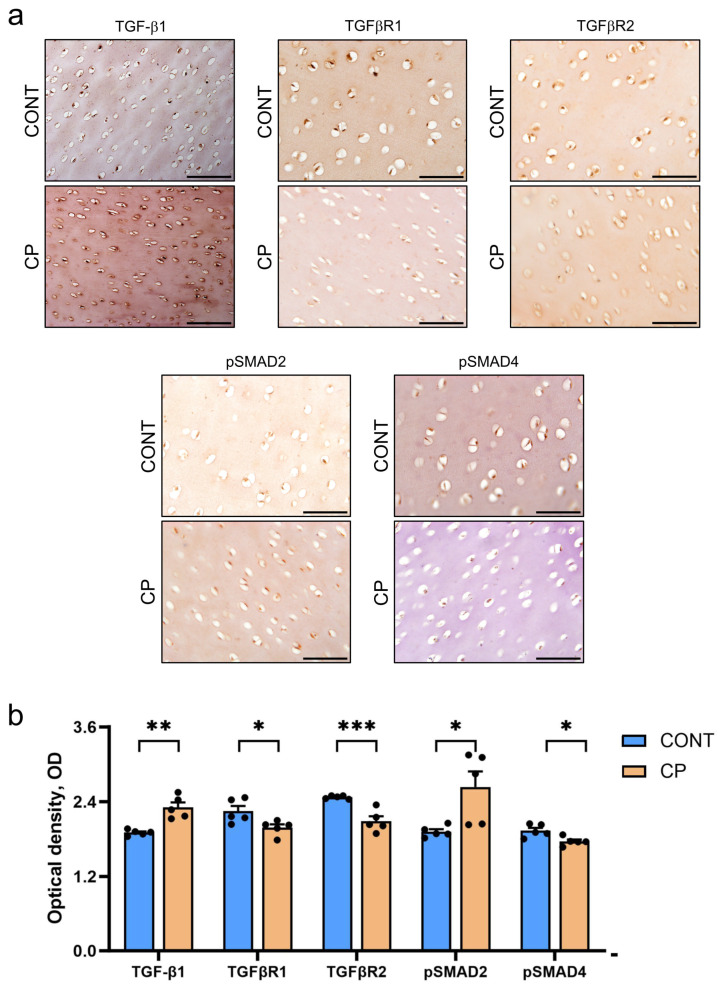
(**a**) Representative immunohistochemical (IHC) staining for TGF-β1, TGFβR1, TGFβR2, pSMAD2, and pSMAD4 in the articular cartilage of the distal femur from control (CONT) and chronic pancreatitis (CP) pigs. Scale bars represent 50 μm. (**b**) Bar graphs illustrating the optical density (OD) of expression for the analyzed proteins. Measurements were conducted on two histological samples per animal in at least ten randomly selected areas. Bars indicate mean values ± standard error (n = 5 pigs per group). Statistical significance is denoted as * *p* < 0.05; ** *p* < 0.01; *** *p* < 0.001.

Figure 6a displays representative images and Figure 6b details the results of the quantitative OD analysis in IHC staining of TGF-β1/mothers against decapentaplegic homolog 2/3 (TGF-β1/SMAD2/3) pathway proteins. The IHC analysis revealed a significant increase in TGF-β1 staining homogeneity in articular cartilage from CP pigs compared to control pigs, where the staining was consistent throughout the cartilage. To further explore the TGFβ pathway, the immunostaining of its receptors I and II (TGFβR1 and TGFβR2, respectively), was conducted. A decrease in the OD of both receptors was observed in the articular cartilage of CP pigs. Additionally, to understand the TGF-β1/SMAD2/3-dependent differences in cell signaling in articular cartilage affected by CP, phosphorylated SMAD2 (pSMAD2) and phosphorylated SMAD4 (pSMAD4) were evaluated. The OD of pSMAD2 immunoreactivity was increased in CP pigs, while pSMAD4 was decreased, indicating differential molecular responses in the TGF-β1/SMAD2/3 pathway due to CP.

### 2.4. Synovial Fluid

**Figure 7:**
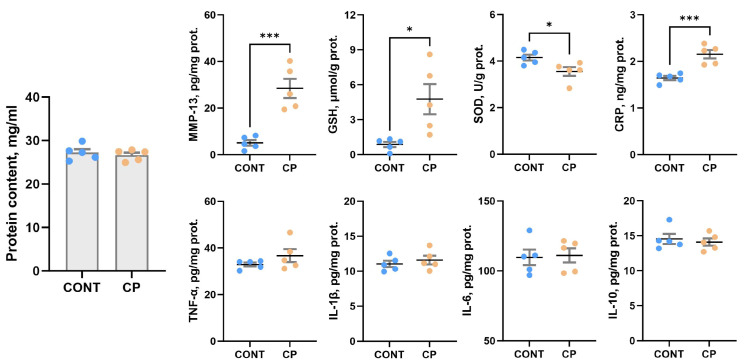
Synovial fluid total protein content and the concentration of synovial fluid MMP-13, selected markers of antioxidant activity (GHD, SOD), CRP and pro- and anti-inflammatory cytokines (TNF-α, IL-1β, IL-6, and IL-10). Each sample underwent three technical replicates for each assay. Plots indicate mean values ± standard error (n = 5 pigs per group). Statistical significance is denoted as * *p* < 0.05; *** *p* < 0.001.

There were no significant differences in the total protein content of the synovial fluid between control piglets and those from the CP experimental group, as shown in Figure 7. Increased C-reactive protein (CRP), reduced glutathione (GSH) and MMP-13 levels, along with a decrease in superoxide dismutase (SOD) activity were observed in the synovial fluid of piglets with CP compared to the control group. CP did not affect CRP concentration, nor the levels of selected pro- and anti-inflammatory cytokines (TNF-α, IL-1β, IL-6, and IL-10) in the synovial fluid.

## 3. Discussion

Previous analysis of all biochemical parameters in the serum of pigs with cerulein-induced chronic pancreatitis (CP) revealed pancreatic and intestinal inflammation, together with an associated increase in the TNF-α concentration in the mucous membrane of the duodenum and pancreas [7]. The trends observed in the data of femoral trabecular bone observed in CP pigs in the current study may be caused by the overall inflammatory state within the body, especially when other specific inflammatory markers like IL-1β, IL-6, and CRP are also elevated [7,25].

A complex network of interactions exists between bone loss (resorption) and inflammatory markers, namely IL-1β, IL-6, TNF-α, and CRP, activating osteoclasts responsible for resorption. The acute-phase protein CRP elevates IL-6 and TNF-α levels, which together enhance the process of bone resorption [18]. Increased levels of IL-6 and CRP suggest general inflammation that also affects the bones and cartilage [17]. IL-1β, IL-6, and TNF-α are interrelated and enhance each other’s effects. IL-1 β stimulates the production of IL-6 [26,27]. An elevated concentration of IL-10 in blood serum could indicate a cellular communication response to an overall low-grade inflammatory state. This response aims to mitigate inflammation, facilitate tissue repair, and prevent immunopathological consequences associated with chronic inflammation [7,28,29].

Intestinal and systemic inflammation, compromising essential nutrient uptake, may lead to malnutrition [30], and a decrease in body weight, diminishing load-related forces on bone tissue which are crucial for maintaining proper bone homeostasis [31].

This is confirmed by the histomorphometrical analysis. The induction of CP by cerulein impacted collagen fibers, resulting in a decrease in loosely packed thin collagen fibers in subchondral bone trabeculae, that indicated disturbances in bone homeostasis and alterations in bone remodeling dynamics caused by systemic inflammation [32,33].

Other factors, over and above general low-grade inflammation in the body, can also affect the body’s ability to maintain normal bone mass. Oxidative stress, which often accompanies inflammation, can lead to the degradation of bone and cartilage tissue [19]. It can affect trabecular bone density, which in turn can increase the risk of fractures and reduce overall bone quality.

Lowered SOD levels and increased GSH levels in the synovial fluid in CP-afflicted animals indicate an adaptive response to oxidative stress and inflammation [34]. Synoviocytes produce synovial fluid, a natural lubricant rich in high-molar-mass hyaluronans, actively contributing to nutrient transport and an anti-inflammatory effect. In cases where inflammation intensifies due to oxidative stress, synoviocyte function is affected, accelerating hyaluronan degradation and contributing to osteoarthritis development [34,35]. The observed decline in SOD activity in pigs with CP suggests heightened cell vulnerability to damage from reactive oxygen species (ROS) produced during inflammatory processes. This increased vulnerability may necessitate higher antioxidant levels to neutralize ROS and prevent cellular damage. Elevated GSH levels indicate an effort to maintain cellular redox balance, aligning with findings from Bolduc et al. [36] and Setti et al. [37]. Li et al. [38] also reported decreased SOD activity in rats with exercise-induced knee OA. However, Xiao et al. [39] observed reduced SOD and GSH in the synovial fluid of mice, but differences may stem from using a distinct arthritis model. Xiao et al. [39] induced arthritis through arthrotomy, while our study involved synovial fluid alterations due to general low-grade inflammation associated with CP, acting as a biochemical stressor.

CRP is a general marker of inflammation and there is a strong positive correlation between synovial and serum CRP levels, although synovial CRP concentration is much lower than that in the blood [40,41]. Therefore, opinions on the usefulness of this marker in diagnostics are divided; some consider CRP to be a good indicator of joint inflammation [42], others do not [41]. Under inflammatory conditions, pro-inflammatory cytokines can affect chondrocytes in articular cartilage, leading to changes in metabolic processes. IL-1β, for example, can stimulate the secretion of cartilage-degrading enzymes such as matrix metalloproteinases (MMPs), leading to the degradation of collagen and proteoglycans in the cartilage matrix [43], while TNFα stimulates resorption and inhibits synthesis of proteoglycans, the main component of the intercellular matrix in cartilage [44]. They enable water retention, enhancing cartilage shock-absorbing and elastic properties. This feature ensures the cartilage’s capability to absorb mechanical forces during joint movement. Proteoglycans are associated with collagen fibers in the intercellular matrix, playing a crucial role in maintaining the structure and integrity of articular cartilage. The current study showed no changes in collagen fibers in articular cartilage and a reduction in proteoglycan content following CP. The main enzyme responsible for tissue remodeling including proteoglycans degradation is MMP-13 [43]. This metallopeptidase, collagenase, is produced by stromal fibroblasts in the promotion of angiogenesis, chondrocytes, synoviocytes, and osteocytes [45]. Although the causative role of osteocytes in joint disease remains unclear, it is known that osteocyte dysfunction in subchondral bone clearly contributes to osteoarthritis. A few hypothetical mechanisms by which subchondral bone deterioration could exacerbate cartilage degeneration and drive articular degeneration have previously been demonstrated. These include biological hypotheses such as cell death or vascular changes, and mechanical hypotheses such as plate distribution in subchondral bone [45]. The perilacunar system connecting osteocytes is important for solute transport, and transport between subchondral bone and cartilage. Any changes within this system contribute to the degeneration of articular cartilage. It has been proven that subchondral bone exhibits perilacunar suppression, collagen fiber disorganization, hypermineralization, and changes in enzyme expression [46]. MMP-13 is the primary MMP involved in cartilage degradation, through its ability to cleave type II collagen [21]. Moreover, IL-1β plays a substantial role in the induction of MMP-13 expression, promoting cartilage matrix degeneration [46]. MMP-13 also targets other matrix molecules such as type I, III, IV, IX, X collagen, osteonectin, perlecan, and proteoglycan, and it is likely involved in matrix turnover in healthy cartilage [21]. MMP-13 proves valuable in the diagnosis and assessment of disease severity, as well as in the prediction of osteoarthritis development. This suggests its potential utility as a biomarker for osteoarthritis [47]. A previous analysis showed increased blood TNF-α concentrations in a pig model of CP [7], whereas in the current study, increased MMP-13 concentrations were observed in the synovial fluid. Together these data indicate that both local and systemic inflammation takes place simultaneously, as was observed in other animal [48] and clinical studies. An increase in the level of MMP-13 in synovial fluid is important in the development and progression of osteoarthritis, even if the expression of MMP-13 in the cartilage itself does not change or only changes slightly in the early stages of development, while the mRNA levels of MMP-13 are increased [47,49]. The unchanged MMP-13 expression observed in the current study may be due to the fact that WB analysis was performed on articular cartilage samples without the calcifying zone [49], while previous studies showed higher MMP-13 activity and expression in calcified cartilage and subchondral bone [50]. MMP-13 increased in the superficial zone of articular cartilage belonged to people with severe stages of osteoarthritis except of synovial membrane [51]. Even though MMP-13 expression did not increase in the articular cartilage of pigs with CP in the current study, the increase in MMP-13 observed in the synovial fluid itself indicates an increased risk of collagen degradation in cartilage tissue, the effects of which could be enhanced by increased MMP-13 in peripheral blood. Moreover, IHC analysis showed a very strong MMP-13 IR signal located in the area of calcification and subchondral bone, which could prove that cartilage degradative changes are not only associated with synovitis but are a secondary response to disturbances in bone homeostasis, which is in line with the mechanism proposed by Chu at al., 2019 [52].

Currently, OA is considered a complex disease, developing locally and involving inflammatory mediators released by joint components such as cartilage, bone, and synovium, which are influenced by a systemic process. The damaged cells of cartilage, bone, and synovial tissue release various pro-inflammatory factors that subsequently stimulate the production of other transmitters with pro-inflammatory and catabolic effects on joint structures, leading to an escalation in the apoptosis of chondrocytes and a reduction in their lifespan [53]. An important element in the etiology of OA is inflammation of the synovium, which maintains the integrity of cartilage tissue by moisturizing it and regulating chondrocyte metabolism [54,55]. OA and synovitis are closely related processes. In damaged cartilage, chondrocytes secrete MMPs and ROS, which, in turn, intensify cartilage damage. This process leads to the release of extracellular matrix degradation products into the joint space. These substances contribute to the initiation of inflammation in the synovium and impact the secretion of secondary inflammatory mediators including TGF-β1 [56]. TGF-β1 plays a critical role in the development, homeostasis, and repair of diverse cartilage-based cellular processes, including proliferation, differentiation, migration, and apoptosis, as well as extracellular matrix (ECM) synthesis and degradation [57]. High concentrations of active TGF-β1 in the subchondral bone of mice are reported to initiate osteoarthritic changes in the cartilage and induce the expression of TGF-β1 in the synovial cells [57]. There is a strong correlation between the expression of TGF-β1 and MMP-13 in OA-affected cartilage. TGF-β can upregulate the levels of MMP13, primarily through the SMAD-independent pathway [57]. TGF-β1 also stimulates MMP13 expression through the activation of runt-related transcription factor 2 (Runx2), via the MAPK/ERK pathway; IL-1β expression through phosphorylated ETS Like-1 (Elk-1) and activating transcription factor (2ATF-2) and activated c-Fos and c-Jun, respectively [58]. Furthermore, TGF-β1 signaling co-operates with BMP signaling to stimulate the anabolic function of chondrocytes, for example, enhancing collagen II (COL2) and aggrecan expression [59].

Our findings align partially with these observations, revealing an 84% increase in TGF-β1 expression in cartilage. It’s important to note a limitation in our study, namely the absence of TGF-β1 determination in the synovial fluid. Nevertheless, IHC analysis confirmed an elevated reaction intensity, consistent with WB results. TGF-β1 plays a crucial role in regulating normal articular cartilage function. Maintaining TGF-β1 within a narrow range is essential, as significant changes in its concentration, regardless of the direction, can lead to abnormal articular cartilage function [60].

The TGF-β1 signaling pathway initiates with the direct binding of TGF-β1 to transmembrane TGFβ type II receptors (TGFβR2). Subsequently, TGFβR2 recruits and activates TGFβR1 (ALK5), which then triggers their intracellular effectors, SMADs. In turn, Smad2 or Smad3 transiently binds to TGFβRI and undergoes activation through TGFβR1-induced phosphorylation in the cytoplasm [61]. By binding to different receptors of the activin receptor-like kinase (ALK) family, TGF-β1 induces intracellular phosphorylation of receptor-regulated SMAD proteins; it phosphorylates SMAD2/3 via TGFβ1R (ALK5) and inhibits the production of catabolic enzymes like matrix metalloproteinases, including MMP-13 [62]. In contrast, phosphorylated SMAD1/5, via ALK1 (or 2 or 3), is essential for chondrocyte hypertrophy and is associated with the expression of matrix metalloproteinase 13 (MMP13), the main cartilage-degrading enzyme. Therefore, balancing TGF-β1 signaling, via SMAD2/3 or SMAD1/5, is important for chondrocytes to maintain cellular homeostasis and deregulation of this balance has been proposed as a cause of OA [62]. In cartilage, TGF-β1 induces pSMAD1/5 and pSMAD2/3, while ALK5 is essential for TGF-β1-induced Smad2/3 [62]. TGF-β1 also blocks the degradation of ECM proteins by increasing the production of protease inhibitors such as TIMP [63]. However, some studies confirm that the inhibition of TGFβ/SMAD signaling is reduced in OA, but others report opposite results and have shown increased TGFβ/SMAD signaling [63,64,65,66]. It depends on the balance between both TGFβ receptors. A predominant ALK5 signaling situation, a shift in receptor balance favoring ALK1 signaling, either by the overexpression of ALK1 or by blocking ALK5, leads to the elevation of MMP-13 [67]. ALK1 signaling apparently stimulates type II collagen degradation via MMP-13, while ALK5 promotes anabolic pathways in chondrocytes, thereby stimulating the synthesis of cartilage matrix molecules [67]. Another intracellular control mechanism of TGFβ signaling involves the inhibition of SMADs, which are critical in the negative feedback regulation of THGβ/BMP signaling [68]. Chondrocytes express the BMP type I receptors ALK2, ALK3, and ALK6 and the type II receptors BMPR2, ACVR2A, and ACVR2B. In both healthy and OA cartilage, *Bmp2* mRNA expression is detected. BMP-2 signaling induces ECM production including proteoglycan content and COL2A1 production [69,70].

The IHC analysis conducted in our study revealed an increase in MMP-13 and TGFβ IR in cartilage due to CP. Interestingly, the immunostaining reaction for TGFβR1, TGFβR2, and pSMAD4 decreased, while pSMAD2 increased in response to CP. These findings suggest that factors beyond the TGF/SMAD2/3 signaling pathway may contribute to MMP-13 production. Notably, MMP-13 synthesis can be influenced by IL-1β and TNF-α, both of which were elevated in the blood of CP pigs, though not in the synovial fluid, and were not assessed by IHC. Additionally, TGFβ signaling collaborates with BMP signaling, and our IHC analysis indicated a decrease in BMP-2, responsible for stimulating chondrocytes to produce collagen, along with reductions in type II collagen and procollagen. The Mankin score indicated mild cartilage degeneration associated with CP, which can be potentially exacerbated by aging and an already existing generalized low-grade inflammation. This is confirmed by the IHC reaction for COMP, a biomarker for cartilage degeneration, associated with osteoarthritis and rheumatoid arthritis [71,72].

This study reveals a multifaceted interplay between chronic pancreatitis (CP) and musculoskeletal alterations in a pig model. The observed changes in subchondral bone and articular cartilage in CP pigs were associated with an overall inflammatory state, as evidenced by elevated levels of pro-inflammatory markers such as IL-1β, IL-6, TNF-α, and CRP [7]. Oxidative stress and alterations in antioxidants in the synovial fluid highlight the impact of inflammation on bone and cartilage health. The study identifies MMP-13 as a potential key player in the development and progression of osteoarthritis, with increased concentrations in synovial fluid indicating both local and systemic inflammation. The complex network involving TGF-β1 signaling, altered receptor expression, and potential shifts in the balance of pro-anabolic and catabolic pathways further underscore the intricate relationship between CP and musculoskeletal changes. Clinical implications include the need for comprehensive assessments of bone health in CP patients and a heightened awareness of potential musculoskeletal complications associated with this pancreatic condition.

While the insights gained from this study are valuable, certain limitations should be noted. Firstly, the sample size may be considered small. Secondly, the study used 2D histological sections instead of micro-CT to determine bone microstructural parameters, which might affect the accuracy of our findings. Additionally, we used optical density measurements, which may not perfectly reflect protein abundances. Moreover, the slight differences in some parameters investigated between the control and CP groups might be due to the early termination time point (6 weeks) of the study. Despite these limitations, we believe that they do not completely undermine the novelty of our study. We assert that conducting analyses on tissues obtained from model animals, which are anatomically and physiologically more similar to humans than traditional laboratory animals, holds significant value for pre-clinical studies in both medicine and bioengineering. This is especially relevant in the context of the current study on CP and its impact on musculoskeletal health, where integrating functional biomaterials tailored for osteoarthritis treatment is crucial. Nevertheless, further studies are recommended for a more comprehensive understanding of protein expression patterns in cartilage and changes in the structure of subchondral bone. The accumulation of such data over time can provide deeper insights into the changes in subchondral bone and articular cartilage associated with CP, which could potentially benefit from the targeted application of biomaterials designed to counteract inflammatory and oxidative stress conditions.

## 4. Materials and Methods

### 4.1. Animals and Experimental Design

The research was carried out on domestic pigs (pbz breed). The study involved 10 uncastrated boars, aged between 9–10 weeks [7]. After a 7-day adaptation period, the pigs were divided into 2 groups: the control group (CONT, n = 5) and pigs subjected to induction of CP (CP, n = 5). Age-, sex- and weight-matched control pigs were used for comparison of the inflammatory systemic and pancreatic changes observed in the CP-pigs. After the adaptation period, CP-pigs received a rapid intramuscular infusion of cerulein (C9026 Caerulein, Sigma-Aldrich Merck KGaA, Darmstadt, Germany), at a dose of 1 µg/kg b.w. for 6 consecutive days, with 24h intervals in between cerulein injections [73]. The animals were then kept for the next 6 weeks. Animals in both groups were fed the same feed mixture, intended for pigs of this age and which contained all nutrients in levels recommended by the National Research Council (NRC) and according to Polish standards of pig nutrition [74,75]. After the end of the experiment (43 days after finishing cerulein injection), blood samples were drawn, and all animals were subjected to pharmacological euthanasia [7].

### 4.2. Sample Collection

Synovial fluid was collected from the joint spaces of knee prior to disarticulation and femur removal. An incision was made at the top of the patella using a scalpel blade. With the knee slightly flexed, a syringe and needle were introduced into the open area below the patella, situated between the femur and tibia. The limb was then manipulated several times through its full range of motion to encourage maximum synovial fluid collection via syringe suction.

Synovial fluid was collected from the joint spaces of both knees prior to disarticulation and femur removal. The collected biofluids (blood and synovial fluid) were centrifuged at 1500× *g* for 15 min. The resulting supernatants were immediately stored at −86 °C following centrifugation.

Samples of distal femoral articular cartilage (from the knee joint) were excised from the left femora and snap-frozen in liquid nitrogen for Western blot analysis. Frontal plane sections encompassing articular cartilage and trabecular bone of the epiphysis and metaphysis were cut from the distal ends of the right femora using a diamond bandsaw. These sections were then fixed in phosphate-buffered paraformaldehyde (4% *v*/*v*, pH 7.0).

### 4.3. Biofluids Parameters

Blood serum and synovial fluid concentrations of MMP-13 were measured using porcine-specific enzyme-linked immunosorbent assay (ELISA) kit from ABclonal (Wuhan, China) for MMP-13 (#RK03352). Additionally, the synovial fluid levels of TNF-α, CRP, IL-1β, IL-6, and IL-10 were determined using commercial porcine-specific ELISA kits from BT-Lab (Shanghai, China) for TNF-α (#E0610Po), CRP (#E0048Po), IL-1β (#E0116Po), IL-6 (#E0122Po), and IL-10 (#E0110Po). Protein concentration in the synovial fluid was determined using the Pierce BCA Protein Assay Kit (ThermoFisher Scientific, Waltham, MA, USA). The synovial fluid concentration of reduced glutathione (GSH) and the activity of total superoxide dismutase (T-SOD) were assessed using commercial kits from Elabscience (Houston, TX, USA) (#E-BC-K030-M and #E-BC-K020-M, respectively). All assays were conducted in accordance with the manufacturers’ protocols, using a Benchmark Plus microplate spectrophotometer (Bio-Rad, Hercules, CA, USA). Each sample underwent three technical replicates for each assay, with all assays demonstrating intra-assay CVs below 8%.

### 4.4. Cartilage Histochemical and Immunohistochemical Analyses

Samples of distal femoral articular cartilage with subchondral bone, after fixation in 10% formalin, were decalcified using Decalcifier II (Leica, Wetzlar, Germany) and then embedded in paraffin. Microtome-cut sections, 4-μm thick, were stained with Safranin-O, according to the protocol described in ref. [76], to analyze basal histomorphometry and proteoglycan content in the articular cartilage matrix, and with Picrosirius red (PSR), according to the protocol described in ref. [77] for subchondral bone histomorphometry and to determine the proportion of thin, immature collagen in the articular cartilage. Stained sections were examined using an Olympus CX43 light microscope (Olympus, Tokyo, Japan). Microscopic images were analyzed using Fiji graphical software (ver. 2.15.0) [78]. Moreover, Mankin scoring system was used to evaluate four key histological aspects of articular cartilage: surface integrity, cellularity, cell cloning, and Safranin-O staining. Each aspect is scored on a scale that reflects the severity of cartilage damage [79], the cumulative score provides a semiquantitative measure of cartilage degeneration.

The histomorphometric analysis of the articular cartilage included measuring the thickness of the superficial, transitional, and deep zones at least ten locations performed on two sections. PSR-stained sections were examined under polarized light to assess subchondral bone histomorphometry and calculate microarchitectural descriptors, including bone volume fraction (BV/TV), trabecular thickness (Tb.Th), trabecular number (Tb.N), trabecular spacing (Tb.Sp), and trabecular fractal dimension. Analyses were performed using the BoneJ plugin (ver. 7.0.17) for ImageJ/Fiji software [80]. The proportion of thin, immature collagen in the articular cartilage, as identified in PSR-stained sections, was quantified as a percentage of the overall collagen content. This quantification was achieved through an automated image analysis utilizing a pixel counting method, facilitated by specifically designed macros in the ImageJ Macro Language (IJM), a scripting language incorporated into Fiji software. Measurements were performed on two histological sections per animal, with four randomly selected areas of the subchondral bone evaluated per section.

Immunohistochemical (IHC) staining was performed on deparaffinized and rehydrated tissue slices according to standard protocols. Endogenous peroxidase activity was blocked using a 3% solution of hydrogen peroxide in deionized water for 10 min. Heat-induced epitope retrieval was carried out in a Rapid Cook pressure cooker (Morphy Richards, Swinton, UK) using Tris-EDTA buffer (pH 9.0). After a 30 min blocking period in blocking serum (UltraVision Protein Block, Thermo Scientific, Fremont, CA, USA), the sections were incubated with porcine-specific antibodies against MMP-13 (#ab75606, Abcam, Cambridge, UK; 1:200 dilution), BMP-2 (#pa5-85956, Invitrogen, ThermoFisher, Waltham, MA, USA; 1:250 dilution), PROCOL-2 (#E-AB-2274, Elabscience, Wuhan, China; 1:200 dilution), COL-2 (#ab34712, Abcam; 1:200 dilution), COMP (#E-AB-14886, Elabscience; 1:200 dilution), TGF-β1 (#orb11468, Biorbyt, Cambridge, UK; 1:200 dilution), TGFβR1 (#AF5347, Affinity Biosciences, Changzhou, China; 1:100 dilution), TGFβR2 (#AF5449, Affinity Biosciences; 1:100 dilution), pSMAD2 (#AF3449, Affinity Biosciences; 1:100 dilution), and pSMAD4 (#AF8316, Affinity Biosciences; 1:100 dilution) diluted in Emerald antibody diluent (Cell Marque Corp., Rocklin, CA, USA). All primary antibodies were incubated overnight at 4 °C. Following this, the sections were processed with the BrightVision detection system (#IM-DPVB110HRP, Immunologic WellMed B.V., Duiven, The Netherlands) according to manufacturer’s protocol. They were developed using DAB (#D5905, Sigma-Aldrich, St. Louis, MO, USA), and counterstained with Mayer’s hematoxylin (Mar-Four, Konstantynów Łódzki, Poland). The IHC images obtained were analyzed using the IHC Profiler plugin [81] compatible with Fiji, employing an optical density (OD) quantitative score for the evaluation of immunoreactivity [82]. In our histomorphometric analysis, the regions of interest (ROIs) were meticulously conducted based on identifiable anatomical landmarks within the layers of the articular cartilage. One ROI, shaped as a rectangle, was specifically sized to one-third of the average total cartilage thickness for all specimens in this study, primarily targeting the radial zone. This proportion was chosen to ensure a comprehensive representation of the cartilage, specifically focusing on areas crucial for understanding cartilage pathology and integrity. The ROIs were placed in 10 distinct, non-overlapping, and randomly selected areas, collectively covering more than half of the total cartilage area in each microscopic image. This approach, facilitated by the ROI Manager function in ImageJ, allowed for consistent and standardized analysis across all samples, accommodating the natural variability and irregularities in cartilage thickness and structure. Measurements were conducted on two histological samples per animal in at least ten randomly selected areas displaying positive immunoreactivity by an associate who was blinded to the treatment groups.

### 4.5. Western Blot Analysis

Total proteins were extracted from briefly pulverized cartilage in liquid nitrogen using ice-cold RIPA lysis buffer with PMSF (#PMSF-RO, Sigma-Aldrich Merck, Darmstadt, Germany) and a protease inhibitor cocktail (#S8820, Sigma-Aldrich Merck). Protein concentration in the supernatants was determined using the Pierce BCA Protein Assay Kit (ThermoFisher Scientific, Waltham, MA, USA). Equal protein amounts were separated by 12% SDS-PAGE and transferred onto Immobilon-P PVDF membranes (Sigma-Aldrich Merck). Blots were blocked in 5% non-fat dry milk in TBS and incubated overnight at 4 °C with primary antibodies: MMP-13 (#ab75606, Abcam, Cambridge, UK; 1:750 dilution), TGF-β1 (#orb11468, Biorbyt, Cambridge, UK; 1:200 dilution), and anti-β-actin antibody (#AF7018, Affinity Biosciences, Changzhou, China; 1:10,000 dilution) for loading control. Alkaline phosphatase-conjugated goat anti-rabbit IgG H&L (#ab97048, Abcam; 1:30,000 dilution) served as the secondary antibody. Immunoreactive proteins were visualized using an alkaline phosphatase procedure with NBT/BCIP (#11681451001, Roche, Basel, Switzerland). Densitometric analysis of protein band intensity, normalized to corresponding β-actin bands, was performed semi-quantitatively using the Band/Peak Quantification plugin in Fiji software [83]. Experiments and analyzes were performed by one technician with two technical replicates for each biological sample.

### 4.6. Statistical Analysis

The statistical analysis of results was carried out using STATISTICA software (ver. 13.3, StatSoft, Inc., Tulsa, OK, USA). Each pig constituted an experimental unit, and measurements were averaged per animal prior to statistical analysis. The Shapiro–Wilk test assessed data normality. Variables with normal distribution were analyzed using the two-sided Student’s *t*-test or Welch’s *t*-test for data with unequal variances, as verified by Levene’s test. Nonparametric data and results of Mankin scoring were evaluated using the Mann–Whitney U test. A *p*-value < 0.05 was considered statistically significant, while values between 0.05 and 0.1 were interpreted as trends towards statistical significance. Graphs were created using GraphPad Prism (ver. 10.1.0, GraphPad Software, San Diego, CA, USA).

## 5. Conclusions

The results of the current study leave no doubt that chronic pancreatitis (CP) in young individuals not only leads to a systemic response, as has already been demonstrated, but may also induce changes in the musculoskeletal system, and could probably be considered a causative factor of chronic non-communicable diseases like OA. Alterations within joints, when manifested later in life, may not be directly attributed to CP. Naturally, further research is warranted to validate the immunohistochemistry (IHC) results and explore the specific cellular signaling pathways most likely implicated in CP-induced OA. Moreover, the role of inflammatory markers, oxidative stress, and their effects on bone and cartilage health elucidated in this research could potentially have implications in broader medical fields, such as rheumatology, orthopedics, and even biomaterials research for osteoarthritis treatment. Understanding these connections may pave the way for interdisciplinary approaches to address both pancreatic and musculoskeletal complications, opening avenues for collaborative research and innovative therapeutic interventions.

## Data Availability

The data presented in this study are available on request from the corresponding author.

## Supplementary Materials

The following supporting information can be downloaded at: https://www.mdpi.com/article/10.3390/ijms25041989/s1.
