# Supplementary material for: Does Chronic Pancreatitis in Growing Pigs Lead to Articular Cartilage Degradation and Alterations in Subchondral Bone?
Source: Int J Mol Sci. 2024 Feb 6;25(4):1989. doi: 10.3390/ijms25041989 (PMC10888541; doi:10.3390/ijms25041989)
Supplement: Supplementary file 1 [file ijms-25-01989-s001.zip › ijms-2804656-supplementary.pdf]

Does Chronic Pancreatitis in Growing Pigs Lead to Articular Cartilage Degradation and Alterations in Subchondral Bone?

Ewa Tomaszewska, Monika Hułas-Stasiak, Piotr Dobrowolski, Małgorzata Świątkiewicz, Siemowit Muszyński, Agnieszka Tomczyk-Warunek, Tomasz Blicharski, Janine Donaldson, Marcin B. Arciszewski, Michał Świetlicki, Iwona Puzio and Joanna Bonior

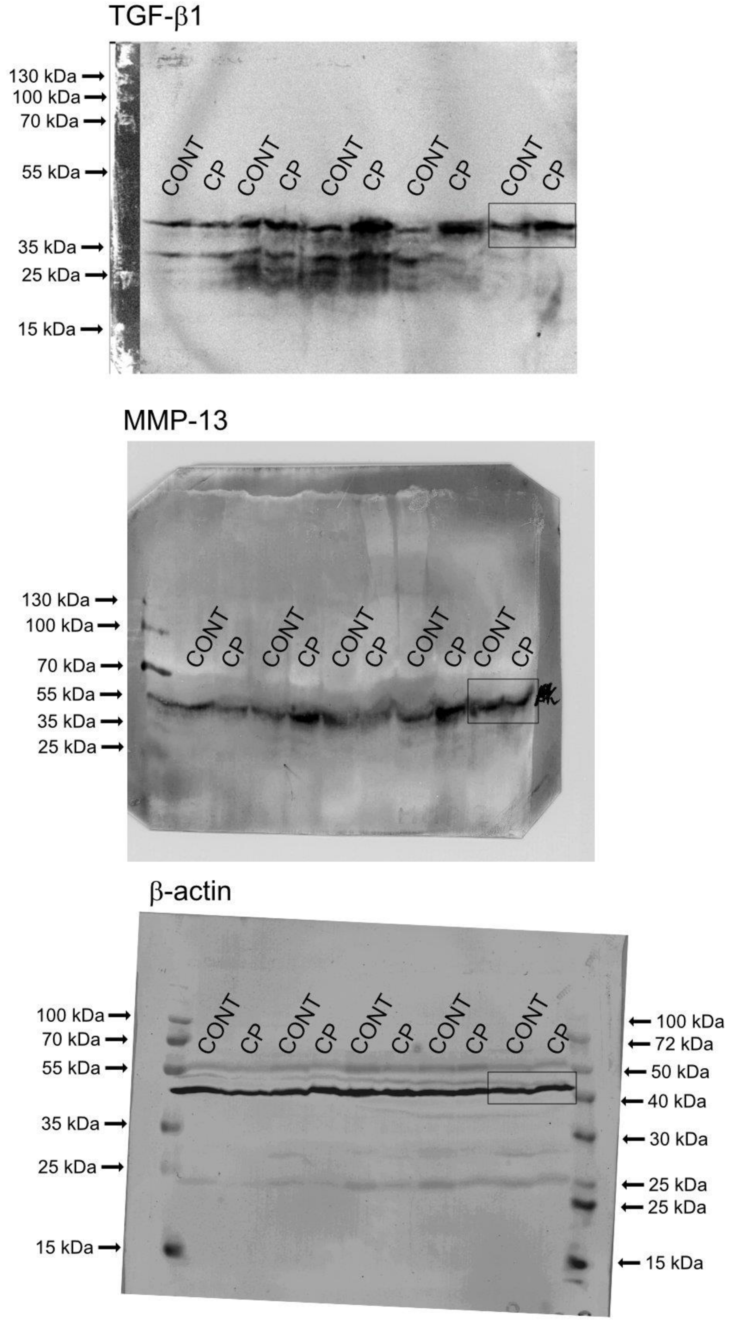

Supplementary Figure S1. Uncropped original Western blot membranes of TGF-β1, MMP-13, and β-actin as loading control (corresponding to Figure 3a in the main article). For all blots, lanes were loaded alternately with subsequent CONT and CP samples Protein ladders used are PageRuler Plus (26619, ThermoFisher, Waltham, MA) for all blots (left), and WideRange Color Protein Ladder (3900-50, AABiot, Gdańsk, Poland) for β-actin (right). Lanes marked with a box were used in Figure 3a in the main article.
